# Relationship between oral microbiota and chronic kidney disease: facts and perspectives

**DOI:** 10.1080/20002297.2026.2687208

**Published:** 2026-06-23

**Authors:** Xinyi Fang, Mengyi Wang, KAIDILIYA Yalikun, Xue Luo, Xiliang Jiang, Moussa Ide Nasser, Chi Liu, Lei Pu

**Affiliations:** a School of Medicine, University of Electronic Science and Technology of China, Chengdu, People's Republic of China; b Department of Stomatology, Sichuan Academy of Medical Sciences· Sichuan Provincial People’s Hospital, Chengdu, People's Republic of China; c Department of Nephrology, Sichuan Clinical Research Center for Kidney Disease, Sichuan Provincial People's Hospital, University of Electronic Science and Technology, Chengdu, People's Republic of China; d Department of Cardiac Surgery, Guangdong Provincial People's Hospital (Guangdong Academy of Medical Sciences), Southern Medical University, Guangdong Cardiovascular Institute, Guangzhou, Guangdong, People's Republic of China

**Keywords:** Oral microbiome, periodontitis, chronic kidney disease, pyelonephritis, pathophysiological mechanisms

## Abstract

**Background:**

The human microbiome comprises the microorganisms inhabiting the body, with the oral cavity representing the second most densely colonized site after the colon. Periodontal disease-associated oral bacteria are more common in patients with kidney disorders than in the general population. Oral dysbiosis disrupts host–microbiota homeostasis and promotes destructive periodontal inflammation, which has been linked to chronic kidney disease (CKD). However, current interventional evidence, including randomized controlled trials, remains limited by small sample sizes, short follow-up, heterogeneous periodontal interventions, inconsistent renal endpoints, limited blinding, and inadequate adjustment for confounders such as smoking, glycemic control, and medication use. Thus, causality between oral microbiota modulation and CKD progression remains unproven.

**Methods:**

This review synthesizes current evidence on the mechanisms by which oral microbiota influence various forms of nephropathy, with a particular focus on the impact of periodontitis (PD) on the progression of renal disease.This review summarizes evidence on mechanisms by which oral microbiota and periodontitis may contribute to nephropathy and renal disease progression.

**Results:**

Oral dysbiosis may affect CKD through systemic inflammation, endothelial dysfunction, and oxidative stress. It may also promote abnormal IgA1 glycosylation in IgA nephropathy and contribute to immune dysregulation and persistent inflammation in glomerulonephritis.

**Conclusion:**

Periodontitis-associated oral dysbiosis may contribute to renal disease pathogenesis and progression. Clarifying these mechanisms could support preventive and therapeutic strategies for patients with nephropathy.

## Introduction

**Figure 1.**
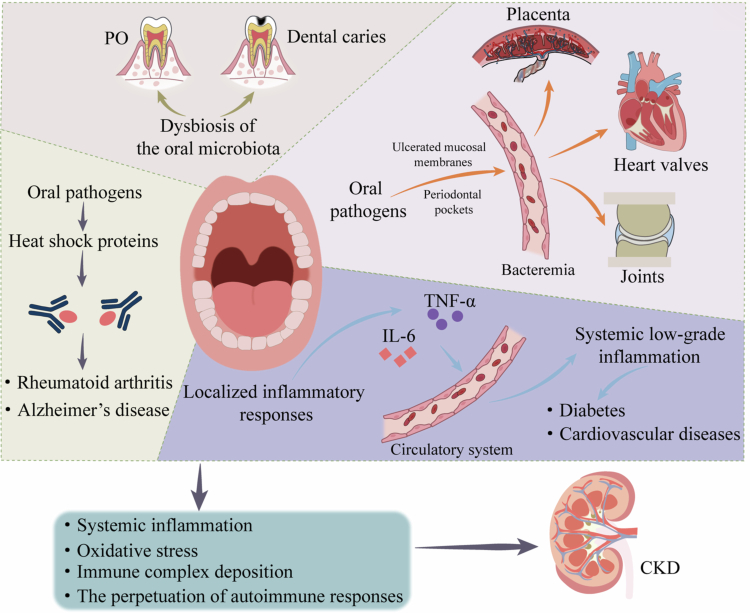
This diagram provides an overview of the intricate relationship between oral microbiota and systemic diseases. (1) Dysbiosis of the oral microbiota may precipitate dental caries and periodontal disease. (2) Oral pathogens, through ulcerated mucosal membranes or periodontal pockets, can enter the bloodstream (bacteremia) and subsequently colonise distant organs, including heart valves, joints, and the placenta. (3) Localised inflammatory responses release cytokines such as IL-6 and TNF-*α*, which, via the circulatory system, instigate systemic low-grade inflammation, thereby exacerbating insulin resistance (diabetes) and promoting atherosclerosis (cardiovascular diseases). (4) Oral pathogens may engage in antigenic mimicry of host proteins (e.g. heat shock proteins), triggering autoimmune responses, as observed in conditions such as rheumatoid arthritis and Alzheimer’s disease. (5) This cascade of events contributes to systemic inflammation, oxidative stress, immune complex deposition, and the perpetuation of autoimmune responses, playing a pivotal role in the pathogenesis of kidney disease.

Dysbiosis of the oral microbiome constitutes a potential pathogenic factor for a variety of diseases; the composition of microbial species and the physical structure of biofilms are closely linked to oral health. Relevant studies have identified alterations in the composition of the oral microbiome associated with an increasing number of diseases and conditions, including diabetes, colorectal cancer, rheumatoid arthritis, and Alzheimer’s disease [1–3](Figure 1).

More than 700 distinct bacterial species have been identified in the oral cavity [4]. These microorganisms are commonly found on the surfaces of our teeth, above the tongue, and in the suspensions and saliva secreted within the oral cavity. A balanced oral microbiome is imperative for maintaining oral health. Microbial communities influence human health and pathological states through a range of biological activities, including the regulation of metabolic processes, energy extraction, defence mechanisms against pathogenic microorganisms, vitamin biosynthesis, and modulation of the immune system [5–8]. The oral microbiome is established through innate transmission from the mother and subsequent acquisition from the environment. The eruption of teeth provides new ecological niches for microorganisms, thereby enhancing their diversity [9,10]. Owing to its nutrient-rich environment, the oral cavity harbours one of the most diverse microbial communities in the human microbiome. Nevertheless, research on the oral microbiome remains relatively underexplored compared with the extensive investigations of the gut microbiome [7,11]. Among these, bacteria are the predominant group, encompassing Gram-positive, Gram-negative, and anaerobic bacteria. Fungi are also common microorganisms in the oral cavity, including Candida species. Other microorganisms present include viruses and mycoplasma, while bacteria such as Streptococcus remain the predominant group.Studies on the oral microbiome have revealed the presence of over 700 distinct bacterial species [4], which predominantly belong to dozens of genera classified within seven phyla, including Actinobacteria (formerly referred to as Actinobacteria [12], Proteobacteria, Saccharibacteria (TM7), and Spirochaetes, among others. The bacterial species constituting the majority of the oral microbiome are typically relatively stable among individuals. However, variations in the relative abundance of taxa, differences at the strain level, and the presence of rare strains and species significantly contribute to the observed genetic diversity among individuals, serving as a means of distinguishing among them [13]. Currently, we recognise that the oral microbiome harbours a substantial number of viruses, as well as less common taxa, such as fungi, protists, and archaea [14,15]. The oral microbiome exhibits a high degree of diversity and a complex ecological framework, in which these communities establish finely tuned, highly structured ecosystems. Within localised niches, they engage in metabolic exchanges shaped by the distinct microenvironments of the oral cavity. The oral microbiome interacts with the human host's immune system, significantly contributing to both oral and systemic health [14]. The oral microbiome is primarily distributed across the oral mucosa, dental surfaces, and periodontal tissues, with the bacterial population present in saliva serving as a particularly representative component of this microbial community. The diversity of the oral microbiome refers to the variety, quantity, distribution, composition, and functional characteristics of the microorganisms present within the oral cavity. In recent years, advances in microbiological research methodologies have yielded a growing body of evidence highlighting a robust correlation between the diversity of the oral microbiome and both oral health and disease [14,15]. The diversity of the oral microbiome is primarily reflected in two aspects: species diversity and population density. At the species level, studies indicate that the oral microbiome comprises three major categories: Gram-positive bacteria, Gram-negative bacteria, and actinobacteria. The most prevalent genera include Bifidobacterium, Streptococcus, Anaerococcus, Lactobacillus, and Staphylococcus. Under normal conditions, the diversity of the oral microbiome remains relatively stable, with the relative abundances of various microbial communities existing in a state of equilibrium. However, when internal or external factors disrupt this balance, it can lead to the onset and progression of oral diseases [15].

**Figure 2.**
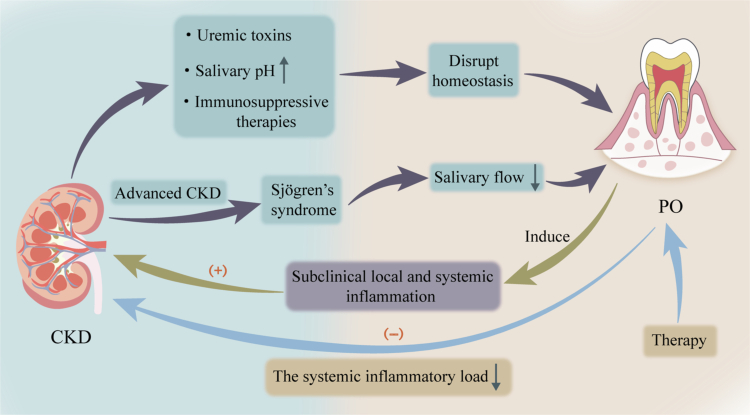
CKD and PD are characterised by a bidirectional relationship. On one hand, the systemic imbalances associated with CKD can predispose individuals to the development of periodontitis, mediated by factors such as uraemic toxins, increased salivary pH, and the effects of immunosuppressive therapies that disrupt homeostasis. Furthermore, Xerostomia, particularly prevalent in advanced CKD patients with Sjögren’s syndrome, leads to a reduction in salivary flow. On the other hand, periodontitis-induced subclinical local and systemic inflammation may exacerbate the progression of CKD. Notably, periodontal therapy has been demonstrated to mitigate the systemic inflammatory load in CKD patients, potentially slowing disease progression.

CKD is defined as an abnormality in kidney structure or function persisting for more than three months, characterised by irreversible loss of nephron units, a decline in glomerular filtration rate (GFR), and an associated increase in end-stage mortality [16]. CKD affects an estimated 700 million individuals worldwide. The spectrum of CKD includes glomerular disorders, vascular nephropathies, tubulointerstitial diseases, cystic kidney disorders, and various other renal pathologies. The predominant aetiological factor for CKD is diabetes mellitus, with hypertension and glomerular disorders constituting the subsequent leading causes [17]. In patients with CKD and PD, C-reactive protein (CRP) levels are significantly elevated, suggesting that CRP may predict the onset of CKD [18,19]. Furthermore, PD has been identified as a non-traditional risk factor for GFR decline [20]. In patients with PD, subgingival bacteria and the host's exaggerated immune response lead to dysbiosis of the oral microbial ecosystem [21]. In patients with advanced CKD, particularly those on dialysis, and/or in individuals with specific etiologies such as Sjögren's syndrome, Xerostomia can significantly impair saliva flow (Figure 2 and Table 1). Circulating bacteria, inflammatory mediators, and/or immune complexes derived from infected or inflamed periodontal tissues exert direct or indirect effects on other body systems, representing key mechanisms that contribute to systemic inflammation [22]. Compelling evidence indicates that chronic inflammation and the pathogenic microbiota associated with PD significantly exacerbate the progression of CKD [23] The quality of the existing evidence varies considerably. Therefore, we summarised the evidence using the GRADE framework (Table 2) to provide readers with a transparent overview.

**Table 1.** Key mechanisms by which oral microbiota contribute to CKD.

Mechanism	Description	Key Molecules or Pathways Involved	Impact on Kidney	References
Immune Dysregulation or Systemic Inflammation	Oral dysbiosis (PD) leads to persistent, low-grade systemic inflammation via bacterial translocation and cytokine release.	TNF-*α*, IL-1β, IL-6, CRP; TLR4/NF-κB pathway; M1/M2 macrophage activation	Promotes glomerular injury, accelerates CKD progression, and increases cardiovascular risk	[18,19,22–33]
Endothelial Dysfunction or Oxidative Stress	Periodontal pathogens (*P*. gingivalis) induce endothelial damage, ROS overproduction, and impair vasoregulation.	ROS, NF-κB, MMPs, TNF-*α*, NO imbalance	Glomerular endothelial injury, oxidative stress, fibrosis, and atherosclerosis exacerbation	[34–42]
Abnormal IgA1 Glycosylation (IgAN)	Oral bacteria (Cnm-positive S. mutans, *P*. gingivalis) alter IgA1 glycosylation, leading to immune complex deposition in glomeruli.	Cnm protein, gingipains, microRNA modulation	IgA deposition in mesangium, proteinuria, haematuria, and IgAN progression	[43–51]
Direct Bacterial Invasion or Renal Infection	Oral pathogens enter the bloodstream via periodontal pockets or mucosal ulcers, colonising the kidneys (pyelonephritis).	Bacteremia, LPS, TLR2/4 activation	Renal infection, inflammation, and potential scarring	[52–55]
Bidirectional Relationship with Periodontitis	CKD exacerbates oral dysbiosis (uremic toxins, Xerostomia); periodontitis worsens CKD inflammation and oxidative stress.	Uremic toxins, salivary pH changes, and inflammatory cytokines	Mutual aggravation of both conditions; higher PO severity in CKD patients	[19,56–63]
Oral–Gut–Kidney Axis	Oral dysbiosis affects gut microbiota and the intestinal barrier, amplifying systemic inflammation that impacts the kidneys.	LPS translocation, gut permeability, systemic cytokines	Enhanced systemic inflammation, accelerated renal damage	[52]

**Table 2.** GRADE evidence quality classification of studies on the association between oral microbiota and CKD.

GRADE Quality	Type of Evidence	Specific Content	References
High	Systematic review/Meta-analysis	The association between CKD and PD	[57]
		Bidirectional relationship between CKD and PD	[19,56]
		The global burden and epidemiology of CKD	[17]
Moderate	RCT	Effect of non‑surgical periodontal therapy on GFR	[64]
	Prospective cohort study	The association between PD and declining renal function in African Americans	[20]
	Cohort study	The impact of PD on kidney disease in patients with type 2 diabetes and end-stage renal disease	[23]
		Oxidative stress in the link between periodontal inflammation and renal function	[36]
		The adverse impact of PD on survival rates in patients with end‑stage renal disease	[29]
		The prevalence and severity of CKD Combined with Severe PD	[58,62,65]
		The association between periodontal status and metabolic syndrome in HD patients	[66,67]
		The association between PD and CKD in Korean and Chinese populations	[68,69]
Very low	Mechanistic study (animal/in vitro)	*P*. gingivalis causes kidney damage through oxidative stress	[70,71]
		S. mutans-induced IgAN-like lesions	[47,48]
	Case series	Cnm-positive S. mutans is associated with IgAN and proteinuria	[44,45]
	Expert opinion/Review	Mechanisms linking oral dysbiosis to systemic diseases	[14,22,25]

## Methods

This article is a narrative review. The databases searched is PubMed, Web of Science, and Google Scholar. The search terms is a combination of keywords and MeSH terms, including oral microbiome, periodontitis, Porphyromonas gingivalis, Streptococcus mutans (S. mutans), chronic kidney disease, IgA nephropathy, glomerulonephritis, and dialysis. No publication date restrictions were applied, but we prioritised studies published from 2000 onward to reflect contemporary understanding of the oral–kidney axis. The search was limited to English‑language articles.

The study selection and inclusion criteria consist of the following three points. First, the original human studies (eg, cohort, case‑control, cross‑sectional, randomised controlled trials) examine associations between oral microbiome/periodontitis and CKD or related renal outcomes. Second, the animal and in vitro mechanistic studies investigated pathways linking oral bacteria to renal pathology. Third, The systematic reviews and meta‑analyses is related to the background.

## The impacts of oral microbiota on oral health

Dental caries is associated with an ecological imbalance within the dental plaque microbiome; the mere presence of bacteria alone does not lead to caries. Bacteria residing in dental plaque exhibit biological characteristics that contribute to the development of dental caries. The pathogenic effects of dental plaque can be summarised as the extensive metabolism of carbohydrates by the bacteria within it, resulting in the production of acidic byproducts that lower the local pH. This acidic environment ultimately leads to the demineralisation of dental hard tissues and the formation of carious lesions (Figure 1) [72,73].

Typically, the bacteria implicated in the development of dental caries must meet three essential criteria: the capacity to form extensive biofilms, the ability to produce organic acids, and a high tolerance to acidic environments. S. mutans is widely recognised as a principal pathogen of dental caries, owing to its possession of the requisite characteristics for caries development, its frequent isolation from carious lesions, and its demonstrated ability to elicit severe pathological outcomes in various animal models [74]. Nonetheless, analyses of cultured microbiomes have revealed that, in many clinical instances, there is neither a significant nor even a minimal presence of S. mutans correlating with the onset of dental caries. Consequently, the assertion that S. mutans is the primary pathogen responsible for dental caries has been called into question [75]. Dental caries is perceived as a consequence of ecological shifts rather than merely an infection by a singular species. While S. mutans is not a prerequisite for the pathogenesis of dental caries, its ability to synthesise extracellular glucans from sucrose is a significant contributing factor in the establishment of dental plaque biofilms and the ensuing ecological dysbiosis [76].

## The bidirectional associations and immune mechanisms between PD and CKD

### The immune connections between PD and CKD

PD is a multifactorial chronic inflammatory condition associated with dysbiosis of the oral microbiome, with prevalence estimates indicating that approximately 50% of adults are affected [77]. It is characterised by gingival inflammation and the irreversible destruction of the periodontium, which includes the cementum, periodontal ligament, and alveolar bone. The sequelae of this condition can lead to tooth loss and contribute to systemic inflammatory processes [78]. Recent research suggests that the onset and progression of periodontal disease are primarily driven by dysbiosis within the symbiotic oral microbiome (dental plaque), which subsequently interacts with the host's immune defences, culminating in inflammation and disease [79] (Figure 1). The mechanisms underlying the association between PD and systemic diseases include the transmission of infection, the dissemination of bacterial toxins, and dysregulation of immune responses [24]. The first two mechanisms suggest that bacteria and their byproducts can translocate from the oral cavity to systemic circulation. The third theory posits that periodontal inflammation may result in systemic damage, with PD defined as inflammatory destruction arising from dysbiosis of the periodontal microbiome. Periodontal tissues are highly vascularises. The continuous flow of gingival crevicular fluid reflects the ongoing inflammatory response, and chemotactic gradients facilitate neutrophil recruitment to the site. This helps maintain a balance between the subgingival microbiota and the host's innate and adaptive immune responses. This dynamic, non-passive relationship that fosters homeostasis is defined as an active state of inflammatory surveillance [25]. Alterations in the microbiome or host homeostasis can precipitate a breakdown of internal equilibrium, resulting in inflammation that ultimately manifests as gingivitis and PD. While gingivitis is initially reversible, inadequate management may lead to the progression to PD, characterised by the destruction of periodontal supporting tissues and irreversible alveolar bone resorption. PD can be systematically classified by severity and stage [26]. Recent comprehensive reviews have encompassed various aspects, including the pathogenesis of the disease, microbial and host biomarkers associated with active sites, and findings from mechanistic studies in animal models of PD [80]. The widely utilised single-cell RNA sequencing (scRNA-seq) methodology has enabled the characterisation of differences between healthy individuals and those with PD. This approach has led to the identification of specific host cell populations that exhibit inflammatory characteristics, enhancing the recruitment of neutrophils and other leucocytes and thereby facilitating antimicrobial defence mechanisms [81]. PD is a microbiota-driven, host-mediated disease that leads to the loss of periodontal attachment and bone. It is associated with elevated systemic inflammatory markers and the presence of comorbidities.

Additionally, periodontal treatment can elicit acute local and systemic inflammatory responses within 24 to 48 hours. This systemic reaction may further complicate the clinical status of patients with incomplete medical histories and/or uncontrolled systemic conditions.

### The associations between PD and kidney disease

According to World Health Organisation statistics, kidney disease ranks as the 14th leading cause of death globally [82]. Infections and atherosclerotic vascular diseases are primary contributors to morbidity and mortality in patients with CKD. Recent findings have established a bidirectional relationship between CKD and periodontal disease [19,56]. A study conducted at the China-Japan Friendship Hospital on patients in the nephrology department undergoing pre-procedural kidney biopsies revealed a significantly higher prevalence of chronic severe PD and aggressive PD among those with IgAN. Furthermore, chronic PD was found to be associated with the onset and progression of IgAN, with patients exhibiting both chronic and aggressive PD presenting with more severe disease manifestations [65]. Given the prevalence of CKD and tooth loss among postmenopausal women, as well as their significant health impact, a recent study identified that postmenopausal women with CKD, particularly those aged 66 to 79, have approximately a 40% likelihood of having fewer than 20 remaining teeth. This underscores the importance of effectively preventing and managing CKD-related disorders of mineral and bone metabolism in postmenopausal women to reduce the risk of tooth loss [83]. Non-surgical periodontal therapy (NSPT) is the preferred and ‘gold standard’ method for treating periodontal infections. It is associated with reduced gingival recession and probing depth. Post-treatment clinical evaluations have shown significant reductions in probing bleeding, decreased levels of inflammatory markers, and alleviation of gingival erythema [84–86]. Additionally, NSPT significantly lowers levels of inflammatory markers and can notably improve renal function parameters [64,85,86]. However, it should be noted that the studies are predominantly small-sample exploratory trials or non-randomised interventions [64,84–86]. Moreover, larger, well-designed RCTs are needed to confirm whether the observed improvements in renal function parameters are directly attributable to periodontal therapy and clinically meaningful in slowing CKD progression. Thus, these findings should be considered hypothesis-generating rather than conclusive.

In addition, CKD exacerbates PD by affecting the oral microenvironment. To begin with, CKD may changes the saliva. Saliva plays a key role in maintaining oral homeostasis through its mechanical cleansing and antibacterial functions. In patients with CKD, particularly those undergoing haemodialyses (HD), saliva flow is significantly reduced [87]. Furthermore, saliva flow increases immediately after dialysis [87]. This suggests that uremic conditions directly affect salivary gland function. This makes CKD patients more susceptible to oral candidiasis, dental caries, and periodontal disease. In addition, CKD may contribute to Xerostomia. In predialysis CKD patients, xerostomia and taste disturbances have been identified as major symptoms directly associated with declining renal function [88]. The aetiology of Xerostomia in CKD patients involves multiple factors and remains incompletely understood. It may be related to fluid intake restrictions, polypharmacy and the direct effects of uremic toxins on the salivary glands. Xerostomia not only impairs quality of life but also weakens saliva’s natural antimicrobial defence, thereby promoting the overgrowth of pathogenic bacteria. Finally, one of the most significant mechanisms by which CKD affects the oral microenvironment is the accumulation of urea and other uremic toxins in saliva [88,89]. As renal function declines, salivary urea concentrations rise significantly, serving as a substrate for urea-producing oral bacteria [88,89]. Bacterial urease hydrolyses urea into ammonia, thereby increasing saliva pH. This rise in pH alters the overall diversity and composition of the oral microbiome [90]. Uremic toxins also impair neutrophil and macrophage function, further weakening the host’s ability to control oral infections.

Through these mechanisms, CKD alters the oral microenvironment, creating a vicious cycle that not only increases the risk of oral diseases but may also exacerbate systemic inflammation, thereby accelerating the progression of CKD [52]. PD exhibits a higher prevalence among dialysis-naïve. CKD patients than in the general population, a phenomenon consistently reported across multiple studies and cohorts [19,57–61]. However, racial disparities may also exist: in the National Health and Nutrition Examination Survey (NHANES) cohort, non-Hispanic Black and Mexican American populations exhibited a higher prevalence of PD among CKD patients, whereas this trend was not observed in non-Hispanic White individuals. Investigations have found that the prevalence of PD increases with the progression of CKD, with higher rates observed in advanced CKD [91,92] than in early stages [62,93]. In dialysis patients, the prevalence of PD exceeds 50% [66,67,94]. However, CKD, defined by a reduced GFR, haematuria, or proteinuria, is more commonly observed among patients with PD across various cohorts [68,69]. In a recent study, Serni et al. identified PD as a prevalent comorbidity in CKD, with reported incidence rates ranging from 34.35% to 93.65% across various studies. The prevalence of PD was particularly pronounced in patients with advanced CKD. In summary, PD manifests more severely in patients with CKD than in the general population, and individuals with greater severity of PD may at an increased risk of further exacerbating the severity of CKD.

## Mechanistic pathways linking oral microbiota to CKD

### Dysregulation of immune responses to pathogenic oral bacteria

**Figure 3.**
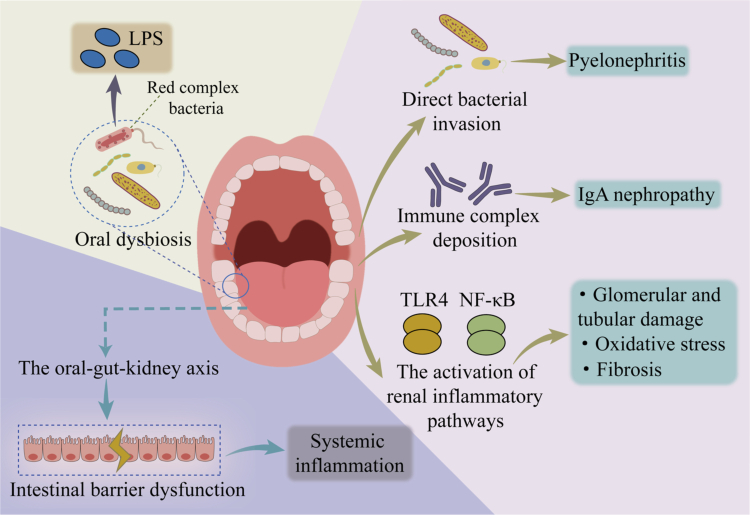
This overview delineates the potential mechanisms by which oral microbiota contribute to renal injury. (1) Oral dysbiosis: The red complex bacteria release virulence factors, including LPS. (2) Renal damage is mediated through mechanisms such as direct bacterial invasion (e.g. pyelonephritis), immune complex deposition (e.g. IgAN), and the activation of renal inflammatory pathways (e.g. TLR4/NF-κB), which culminate in glomerular and tubular damage, oxidative stress, and fibrosis. Furthermore, the oral-gut-kidney axis (depicted by the dashed arrow) exacerbates systemic inflammation by disrupting intestinal barrier function. The interaction between periodontitis and CKD progression is emphasised, with molecular mediators such as TNF-*α*, IL-1β, and C3a playing pivotal roles in the pathophysiology.

In pyelonephritis, pro-inflammatory cytokines such as tumour necrosis factor-alpha (TNF-*α*), monocyte chemoattractant protein-1 (MCP-1), interleukin-6 (IL-6), interleukin-8 (IL-8), and interleukin-23 (IL-23) play a crucial role in initiating the immune response [53,54]. The kidney's initial response to injury or infection resembles that of most organs. During the infectious process, interleukin-1 (IL-1) activates the expression of adhesion molecules in endothelial cells and induces the expression of additional chemokines to recruit leucocytes (WBCs). TNF-*α* further activates the endothelial inflammatory response, resulting in capillary leakage of infiltrating immune cells. Monocytes and macrophages are subsequently recruited to the site of inflammation by MCP-1. During this period, elevated IL-6 levels induce fever and the acute-phase protein response. Neutrophils are recruited to the site of inflammation through chemotactic signalling by IL-8, while IL-23 promotes the proliferation of Th17 cells, thereby intensifying the pro-inflammatory response [55]. Pyelonephritis is an acute infection typically caused by intestinal bacteria such as E. coli. In contrast, the immune dysregulation associated with periodontitis is a chronic, low-grade inflammatory process that gradually leads to the progression of CKD. In PD, there is an interaction between the periodontal microbiome and the host's inflammatory response. However, the mechanisms by which PD may influence CKD progression remain unclear [27–29]. Dysregulated inflammatory responses may arise from the effects of specific pathogens associated with dysbiosis in the PD microbiome, but whether this exacerbates CKD remains to be determined [27–29]. Three microorganisms closely related to PD are Bacteroides fragilis, Treponema denticola, and Porphyromonas gingivalis (*P*. gingivalis). All of these have been linked to elevated antibody titre in patients with CKD [70,71,95]. *P*. gingivalis is associated with immune dysregulation by interfering with macrophage signalling and promoting sustained inflammation. Its fimbrial proteins activate complement receptor 3 on monocytes, which may lead to decreased production of interleukin-12 (IL-12) and interferon-gamma (IFN-*γ*), thereby impairing macrophage function and prolonging inflammation by hindering bacterial clearance. Additionally, lipopolysaccharides (LPS) from Gram-negative bacteria, such as *P*. gingivalis, induce renal fibrosis by elevating transforming growth factor-beta (TGF-*β*) expression in the renal cortex (Figure 3). The LPS-activated Toll-like receptor (TLR) signalling pathway mediates the production of TNF-*α* and IL-6 in glomerular endothelial cells through the activation of M1 and M2 macrophages, thereby affecting renal function and promoting glomerulosclerosis (Figure 3 and Table 3) [30,31]. Another significant virulence factor of *P*. gingivalis is a family of cysteine proteases known as gingipains. Gingipains contribute to renal damage by mediating inflammatory responses that involve various immune cells, including neutrophils, macrophages, and T cells. Gingipains facilitate immune evasion by engaging the TLR 2-phosphoinositide 3-kinase signalling pathway, thereby allowing the bacteria to evade phagocytosis. Additionally, gingipains induce interleukin-17 (IL-17) expression, contributing to renal fibrosis (Figure 3 and Table 3) [70].

**Table 3.** Association between specific oral pathogens and kidney diseases.

Oral pathogen	Associated oral disease	Linked kidney disease	Proposed mechanism	References
P. gingivalis	Periodontitis (red complex)	CKD progression, glomerulosclerosis, IgAN	LPS/TLR4 activation, ROS production, immune evasion via gingipains, increased TGF-*β*, renal fibrosis	[30,31,49,70,71,95]
Cnm-positive S. mutans	Dental caries	IgAN	Cnm alters IgA1 glycosylation; immune complex deposition	[43,44,46–48]
Treponema denticola	Periodontitis (red complex)	CKD (elevated antibody titre)	Immune dysregulation, persistent inflammation	[70,71,95]
Campylobacter rectus	Periodontitis	IgAN (proteinuria)	Correlation with proteinuria in IgAN patients	[45]
Aggregatibacter actinomycetemcomitans	Periodontitis	IgAN remission post-treatment	Associated with reduced proteinuria/haematuria after tonsillectomy + steroids	[50]
Fusobacterium nucleatum	Periodontitis	IgAN remission post-treatment	Similar to above, it suggests a role in immune modulation	[50]
Oral microbiota dysbiosis (general)	Periodontitis, caries	Pyelonephritis, glomerulonephritis, CKD	Bacteremia, cytokine storm (TNF-*α*, IL-6, IL-8), and endothelial dysfunction	[32,33,52–55,96]

### Dysbiosis of the oral microbiome leads to persistent inflammation and CKD progression

Alterations in the oral microbiome can impact distal organs through multiple mechanisms, including translocation, bacteremia, or the toxic effects of bacterial compounds [52] (Figure 1). However, one proposed mechanism is likely mediated by the immune system and inflammatory responses. One hypothesis regarding the role of oral microbiota in kidney disease is that streptococcal infections can trigger glomerulonephritis. However, the more subtle, persistent, and interconnected influence of the oral microbiome on the immune system in patients with CKD may be a significant factor in promoting systemic inflammation. CKD is characterised by a pro-inflammatory state, as evidenced by elevated levels of inflammatory markers, including cytokines. Numerous lines of evidence support the direct pathogenic role of inflammation in the progression of CKD, and it has been observed that increased mortality in CKD is associated with the development of various complications, such as malnutrition, coronary calcification, atherosclerosis, and cardiovascular disease (Figure 1). In CKD, pro-inflammatory mediators, including IL-6, TNF-*α*, adhesion molecules, and adipokines, are elevated (Figure 1). These molecules contribute to the systemic inflammatory response and are implicated in the pathophysiological progression of CKD [32,33]. In another article, a relationship between systemic low-grade inflammation and PD was established; elevated levels of systemic pro-inflammatory cytokines, such as interleukins IL-1 and IL-6, TNF-*α*, and adhesion molecules, correlate with the prevalence and severity of PD [96](Figure 1). Research has shown a close association between the presence of periodontal bacteria and serum TNF-*α* levels, which can predict the extent of renal impairment, as reflected in GFR, and periodontal conditions such as plaque index, gingival index, probing depth, or clinical attachment level (CAL) (Figure 1). In patients with PD, the standard oral microbiome is altered to favour the survival of periodontal pathogens, including Aggregatibacter actinomycetemcomitans, Fusobacterium nucleatum, Tannerella forsythia, *P*. gingivalis, Prevotella intermedia, and Treponema denticola. These microorganisms, directly and indirectly, contribute to bacterial-induced periodontal destruction, forming the ‘red complex’ pathogenesis model [95]. Keystone pathogens are species that exert a disproportionate impact on their communities relative to their abundance [97]. They can evade immune responses through various mechanisms, inducing prolonged inflammation that affects multiple organs, including the kidneys (Figure 1). In particular, *P*. gingivalis, a key pathogen that initiates the subgingival microbiome, undermines the local immune system by evading and damaging components of the host immune-inflammatory response, thereby altering the growth and development of the entire subgingival biofilm. LPS primarily activates TLRs via MyD88 (myeloid differentiation primary response gene 88), leading to the transcription of pro-inflammatory cytokines, mediated by nuclear factor kappa B (NF-κB). This mechanism facilitates the recruitment of inflammatory cells from the adaptive immune system, aiming to mitigate or resolve inflammation. However, other intracellular signalling cascades, such as those mediated by phosphoinositide 3-kinase (PI3K) or complement factor 5 (C5)-induced cyclic adenosine monophosphate (cAMP) [71], may disrupt the normal activation of phagolysosomes in macrophages and neutrophils, leading to macrophage immune suppression and enhanced pathogen survival. Therefore, bacteria may evade immune clearance, potentially leading to persistent, inefficient inflammation that has been suggested to transform normal homoeostatic interactions between the host and the microbiome into a pathogenic relationship. LPS-mediated activation of the innate immune system may also exert systemic effects on distant organs, such as the kidneys. In diabetic patients, *P*. gingivalis can translocate into the gingival biofilm and interact with endothelial cells, which express TLR2 and TLR4. This interaction activates endothelial cells and upregulates adhesion molecules such as VCAM-1 and E-selectin. Consequently, this process results in leucocyte margination and inflammation of the glomeruli and interstitial tissues (Figure 3 and Table 3) [98]. On the other hand, the microenvironmental changes induced by CKD also impair both innate and adaptive immunity. In CKD, the antigen-presenting capabilities of dendritic cells and macrophages are diminished, leading to reduced monocyte stimulation efficiency. Additionally, neutrophil phagocytic capacity is impaired, leading to decreased cytokine secretion. One reason for persistent, chronic inflammation is the immune system's inefficiency, as observed in PD. A key factor is the reduced expression of TLR4 in predialysis and HD patients, particularly in those prone to infections. This diminished TLR4 expression is associated with decreased synthesis of TNF-*α*, IL-1, IL-6, and IL-8 in response to LPS stimulation [99]. In HD patients, research has demonstrated that endotoxins in dialysis fluid may reduce TLR4 expression [100]. This decline is likely a critical factor contributing to the dysfunction of antigen-presenting cells and the heightened susceptibility to infections observed in this patient population.

### Endothelial dysfunction and oxidative stress (CKD)

This dysbiosis is further exacerbated by aberrant production of pro-inflammatory cytokines, chemokines, and metalloproteinases, as well as increased osteoclast activity and immune cell recruitment [34]. The significantly increased mortality rate observed in individuals with CKD is primarily attributable to cardiovascular disease [101]. PD is associated with numerous chronic conditions, particularly diabetes mellitus, pregnancy-related complications, and cardiovascular diseases [79]. Systemic inflammation and protein catabolism accelerate the development of atherosclerosis, a phenomenon referred to as the malnutrition-inflammation-atherosclerosis syndrome [102]. This syndrome is a significant factor contributing to atherosclerosis progression, as well as the increased cardiovascular morbidity and mortality in patients with CKD. It represents a crucial factor in the progression of atherosclerosis, as well as the increased cardiovascular morbidity and mortality observed in individuals with CKD. Furthermore, oral bacteria associated with PD are more prevalent in patients with CKD than in the general population. When patients have concurrent CKD and PD, the severity of PD is significantly greater in CKD patients compared to the general population [62]. The seriousness of PD is positively correlated with the severity of CKD [58,63]. TNF-*α* plays a pivotal role in the progression of glomerular diseases, such as the inflammatory responses observed in diabetic nephropathy. Notably, it has been reported that TNF-*α* levels are significantly elevated in patients with PD who also have CKD [18]. Matrix metalloproteinases (MMPs) are a group of enzymes involved in tissue repair and apoptosis, some of which are upregulated during periodontal inflammation [35]. In the kidneys, MMPs are implicated in the progression of chronic fibrosis and CKD. Therefore, the systemic overexpression of MMPs induced by PD may contribute to renal injury [35]. An extensive longitudinal cohort analysis suggests that CKD and PD are reciprocal risk factors, with oxidative stress serving as a significant mechanism of their interaction [36].

The kidneys receive approximately 25% of cardiac output to sustain glomerular filtration, and PD has been reported to be associated with alterations in endothelial regulation of oxidative stress, vasoconstriction/dilation, platelet aggregation, and leucocyte adhesion. Such alterations evidently affect glomerular endothelial function [37,38].

*P*. gingivalis, a key pathogen in PD, can be detected in various endothelial cells; it triggers an increase in reactive oxygen species (ROS), followed by NF-κB-mediated inflammation, multinucleated adhesion, and apoptosis. An essential defensive mechanism against the bacterial DNA of PD is the production of ROS by leucocytes during the inflammatory process [38]. However, excessive inflammation induced by the aforementioned evasion mechanisms can lead to overproduction of ROS, resulting in a systemic imbalance between pro-oxidants and antioxidants and potentially harming various organs, including the kidneys. Thus, PD may contribute to oxidative stress, and elevated oxidative stress is a significant characteristic of CKD, further emphasising the link between PD and CKD. Research indicates that certain systemic oxidative stress markers, such as serum 4-hydroxynonenal (4-HNE), are associated with PD severity [39]. A study revealed that patients with both CKD and PD exhibited significantly higher levels of glutathione oxidative stress markers than those with either CKD or PD alone, and these levels correlated with periodontal parameters. This finding suggests that CKD and PD are interrelated risk factors, with oxidative stress as one underlying mechanism (Table 3).

PD also affects the balance between vasodilation and vasoconstriction; endothelial dysfunction, as measured by brachial artery flow-mediated vasodilation, is associated with PD and various inflammatory markers [40]. Oxidative stress is commonly observed in patients with CKD and those undergoing dialysis; thus, it is currently considered a non-traditional risk factor for mortality in CKD patients [41,42]. ROS further amplifies the inflammatory response by triggering pro-inflammatory mediators, including NF-κB-related cascades. In the kidneys, oxidative stress can lead to progressive renal damage, glomerulosclerosis, and interstitial fibrosis, exacerbating the already ongoing severe inflammatory processes.

## Abnormal glycosylation of IgA1 and immune complex deposition in IgAN

Oral bacteria, particularly collagen-binding protein (Cnm) ‑positive S. mutans and certain periodontopathogens, can induce abnormal glycosylation of IgA1, leading to the deposition of immune complexes. IgAN is characterised by the deposition of IgA in the mesangial region of the glomerulus, with the IgA subclass being predominantly IgA1. This IgA is thought to originate from mucosal areas of the upper respiratory tract, including the tonsils or oral mucosal tissues, collectively referred to as nasopharyngeal-associated lymphoid tissue (NALT)11.

S. mutans is the primary pathogen responsible for dental caries [103]. Approximately 10–15% of S. mutans strains isolated from the oral cavity exhibit collagen-binding properties due to the presence of a 120 kDa Cnm encoded by the cnm gene [43,44].

Cnm-positive S. mutans has been linked to several diseases, such as infective endocarditis [104,105] and exacerbated cerebral haemorrhage [106–108]. Recent studies have also suggested that Cnm-positive S. mutans strains are associated with the virulence of IgAN [43–48]. A study found that the group infected with Cnm-positive S. mutans exhibited significantly higher caries prevalence and urine protein levels than the Cnm-negative S. mutans group, suggesting a correlation among Cnm positivity, caries status, and urine protein levels in IgAN patients [44].

A study suggests that the unknown effects of the Cnm may exacerbate IgAN by driving immune responses in the tonsils, as the protein is continuously supplied to the tonsils via saliva [46]. The detection rate of *P*. gingivalis, a periodontal bacterium in the red complex, is significantly higher in IgAN patients than in those with chronic tonsillitis [49]. Oral bacteria associated with PD, such as Campylobacter rectus, have also been shown to be significantly correlated with proteinuria in IgAN patients [45]. Additionally, Actinobacillus actinomycetemcomitans and Fusobacterium nucleatum are strongly associated with remission of proteinuria and haematuria induced by tonsillectomy and corticosteroid pulse therapy [50], suggesting that various oral bacteria may be involved in the pathogenesis of IgAN [45].

Oral bacteria, such as Cnm-positive S. mutans and periodontal pathogens, can modulate microRNAs, altering IgA1 production and, in some cases, leading to abnormal IgA1 glycosylation. This abnormal glycosylation of IgA1 induces the formation of anti-glycosylated IgA-IgG complexes [51]. These immune complexes of abnormal glycosylated IgA1 and IgA-IgG deposit in the mesangial region of the glomerulus, leading to the development of IgAN. Chronic oral bacterial infections, such as those caused by Cnm-positive S. mutans and periodontal bacteria, may thus play a role in the pathogenesis of IgAN.

## Clinical implications and prospects for translational medicine

A growing body of evidence suggests an association between PD and the progression of CKD. This is not only of significant clinical importance but also highlights several critical knowledge gaps.

Given the significantly higher prevalence of PD among CKD patients and the association between severe PD and more advanced stages of CKD, routine periodontal screening should be considered an essential component of multidisciplinary care for CKD patients [57]. Dental professionals can perform simple examinations, such as assessing gingival bleeding, measuring periodontal pocket depth, and evaluating tooth mobility. If abnormalities are detected, patients should be referred for a comprehensive periodontal evaluation. In nephrology clinics, asking patients about symptoms such as gingival bleeding, halitosis, or tooth mobility can serve as a preliminary screening tool.

Furthermore, establishing a two-way referral system between nephrology and dentistry is crucial. Patients newly diagnosed with moderate-to-severe CKD (stages 3–5) should be referred for an oral health assessment, particularly those with poorly controlled diabetes or hypertension. Conversely, patients with severe PD and risk factors for kidney disease (such as diabetes, hypertension, or proteinuria) may benefit from renal function screening. Such collaboration is particularly important prior to kidney transplantation, as untreated PD may serve as a source of post-transplant infection.

Attention should be paid to the potential role of periodontal treatment in maintaining renal function. Studies have shown that NSPT can reduce levels of systemic inflammatory markers [109]. Moreover, in some exploratory studies, it may improve renal function indicators, such as GFR and proteinuria [110]. Although these findings are encouraging, the current evidence is limited by small sample sizes, short follow-up periods, and the lack of sham-surgery control designs. Nevertheless, with appropriate antibiotic prophylaxis, periodontal treatment is safe for patients with CKD, particularly those with established vascular access or artificial heart valves. Improved oral hygiene also helps reduce the overall inflammatory burden. And inflammation has been identified as a factor driving the progression of CKD. Therefore, until confirmed by larger-scale trials, periodontal treatment should be viewed not only as a means of maintaining oral health but also as a potential renal protective intervention.

However, we must recognise that there are currently no large-scale RCTs demonstrating that periodontal treatment can slow the decline in GFR or reduce the incidence of end-stage renal disease [111]. Most existing intervention studies have used surrogate endpoints (inflammatory markers or short-term changes in GFR). Apart from that, sample sizes have been insufficient to detect hard renal outcomes. Furthermore, the heterogeneity of periodontal treatment protocols, concomitant medications, and patient populations limits the generalisability of the findings. Until definitive RCTs are completed, clinical decisions regarding periodontal treatment for patients with CKD should be based primarily on oral health needs, while acknowledging that any potential renal benefits remain speculative.

In addition, a major methodological limitation of the current evidence must be acknowledged. Many of the cited studies focus on specific pathogens or serological markers rather than on the oral microbiome as a whole. Therefore, conclusions referring to the ‘oral microbiome’ may be methodologically overstated. There is a fundamental distinction between older pathogen-focused studies and modern comprehensive microbiome analyses that assess community composition, diversity, and functional potential. Future research should employ unbiased, high-throughput sequencing to determine whether global oral dysbiosis, rather than isolated pathogens, contributes to CKD progression.

## Conclusion and future perspectives

Growing evidence from observational studies indicates that disruption of oral microbiome homeostasis, leading to PD, may contribute to kidney disease through mechanisms involving persistent, ineffective inflammation, oxidative stress, and endothelial dysfunction. Interventional studies, including small-scale RCTs, have reported improvements in renal parameters following non-surgical periodontal therapy. However, these findings are limited by methodological shortcomings, including small sample sizes, short follow-up, lack of blinding, and heterogeneity in intervention protocols. The overall quality of current evidence, when assessed using GRADE criteria, is low to moderate. Therefore, definitive causal conclusions cannot be drawn. Future well-powered, sham-controlled, multi-centre RCTs with long-term follow-up and standardised renal outcome measures are essential to determine whether periodontal therapy can prevent or slow the progression of chronic kidney disease. Moreover to elucidate the immunoregulatory mechanisms by which the oral microbiome influences kidney disease pathogenesis.

## Data Availability

Not applicable.
